# Effect of SiC Nanoparticles on AZ31 Magnesium Alloy

**DOI:** 10.3390/ma15031004

**Published:** 2022-01-28

**Authors:** Murugan Subramani, Song-Jeng Huang, Konstantin Borodianskiy

**Affiliations:** 1 Department of Mechanical Engineering, National Taiwan University of Science and Technology, Taipei 10607, Taiwan; smurugan2594@gmail.com (M.S.); sgjghuang@mail.ntust.edu.tw (S.-J.H.); 2 Department of Chemical Engineering, Ariel University, Ariel 40700, Israel

**Keywords:** AZ31 magnesium alloy, nanocomposites, stir casting, mechanical properties, wear resistance

## Abstract

Magnesium alloys are attractive for the production of lightweight parts in modern automobile and aerospace industries due to their advanced properties. Their mechanical properties are usually enhanced by the incorporation with reinforcement particles. In the current study, reinforced AZ31 magnesium alloy was fabricated through the addition of bulk Al and the incorporation of SiC nanoparticles using a stir casting process to obtain AZ31-SiC nanocomposites. Scanning electron microscope (SEM) investigations revealed the formation of Mg_17_Al_12_ lamellar intermetallic structures and SiC clusters in the nanocomposites. Energy dispersive spectroscopy (EDS) detected the uniform distribution of SiC nanoparticles in the AZ31-SiC nanocomposites. Enhancements in hardness and yield strength (YS) were detected in the fabricated nanocomposites. This behavior was referred to a joint strengthening mechanisms which showed matrix-reinforcement coefficient of thermal expansion (CTE) and elastic modulus mismatches, Orowan strengthening, and load transfer mechanism. The mechanical properties and wear resistance were gradually increased with an increase in SiC content in the nanocomposite. The maximum values were obtained from nanocomposites containing 1 wt% of SiC (AZ31-1SiC). AZ31-1SiC nanocomposite YS and hardness were improved by 27% and 30%, respectively, compared to AZ31 alloy. This nanocomposite also exhibited the highest wear resistance; its wear mass loss and depth of the worn surface decreased by 26% and 15%, respectively, compared to AZ31 alloy.

## 1. Introduction

Magnesium and its alloys are attractive to modern industries, mostly due to their low density compared with traditional structural metals, such as aluminum and steel. Additionally, these alloys also exhibit a high strength-to-weight ratio; therefore, they generate significant interest for application in various structural applications in industries, such as automotive, aerospace, and marine industries [1,2,3]. However, Mg alloys have some limitations, such as a poor creep and wear resistance, and low ductility, which is attributed to the hexagonal closed-packed (HCP) crystal structure of Mg, with a limited number of slip systems [4,5]. Certain limitations can be overcome with addition of harder ceramic reinforcement particles to produce metal matrix composites (MMCs) [6,7].

Usually, light-weight materials reinforced using ceramic particles, such as SiC, Al_2_O_3_, TiB_2_, carbon nanotubes (CNT), and others, are used to produce MMCs [8,9,10,11,12,13]. Among these particles, SiC is the most applicable reinforcement for aluminum and magnesium alloys due to its high strength and hardness, low cost, and good compatibility with the metallic matrix. Moreover, several works on Mg composite production with incorporated SiC particles showed enhanced anti-corrosion properties and a high wear resistance [4,14]. Other works showed the addition of aluminum in magnesium alloys to achieve an improvement in the hardness, strength, and wear resistance of MMCs [15]. Incorporation of nanoparticles in magnesium and its alloys demonstrated the formation of more stable secondary phases and a reduction in ductility. Therefore, nanoparticles should be substituted, instead of microparticles, in magnesium and aluminum alloys [16]. Wang et al. investigated the effects of SiC nanoparticles on the microstructural modifications and strength of AZ31 magnesium alloy [17]. Authors have revealed that the AZ31-SiC nanocomposite has a significantly improved strength, and that it has a more lamellar structure compared with unreinforced composites. Han et al. reported the effect of Al and Al_2_O_3_ on the mechanical properties of AZ31 magnesium alloy [15]. Their results revealed that the presence of Al and Al_2_O_3_ assisted in enhancing the mechanical properties, such as hardness, ductility, tensile strength, and work of fracture. Khosroshahi et al. studied the effects of incorporation of SiC nanoparticles into AZ80 Mg cast alloy [18]. The authors found that SiC nanoparticles are advantageous in terms of strength and ductility in comparison with combinations of TiO_2_ and Al_2_O_3_ particles. The wear properties of magnesium MMCs reinforced with SiC particles were reported by Pessato et al. [19]; the authors also reported a higher wear resistance obtained for an AZ91D/SiC composite.

Various fabrication methods are available for manufacturing high-performance MMCs. Stir casting is one of the commonly accepted and economical routes that is practiced commercially due to its advantages, such as mass production, low processing cost, and adaptability. The fabrication of nanocomposites is challenging in terms of attaining a uniform distribution of nanoparticles and a minimization in porosity [4,6,20].

In the current work, a study on the incorporation of SiC nanoparticles into magnesium alloy using a stir casting method and the obtaining of uniformly distributed nanoparticles in a nanocomposite, was carried out. To best of our knowledge, works on nanocomposite production using stir casting methods are very rare. Here, a fabrication method for nanocomposite AZ31-SiC using a stir casting method is demonstrated. Microstructural evaluations, as well as the mechanical properties, hardness, and wear resistance at room temperature, were investigated and the obtained results are discussed.

## 2. Materials and Methods

**Figure 1:**
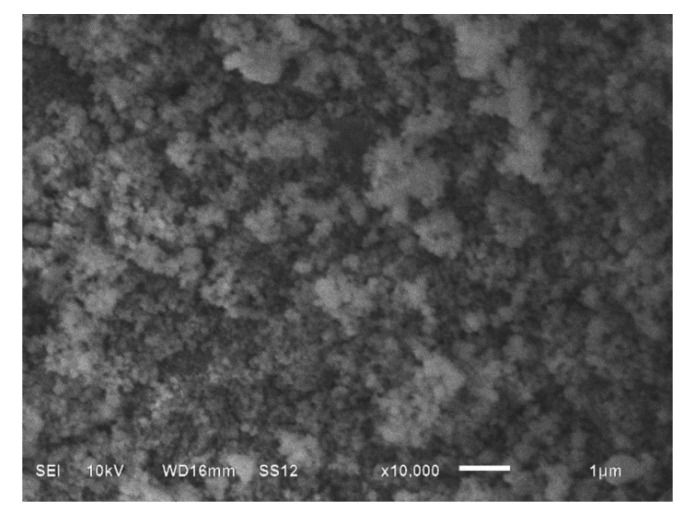
SEM image of commercial SiC nanoparticles.

Commercial AZ31 Mg alloy was used as the matrix component, and was composed of Al—2.95 wt%, Zn—0.64 wt%, Mn—0.26 wt%, Fe—0.005 wt%, and Mg—balance (Kuangyue Technology Co., Ltd., Taipei, Taiwan). Reinforcements of SiC nanoparticles, produced using the plasma arc vapor method, with an average size of 60 nm (IoLiTec Ionic Liquids Technologies GmbH, Heilbronn, Germany), were used in this work. These nanoparticles were examined using a JSM 6510LV scanning electron microscope (SEM, Jeol, Tokyo, Japan) and the obtained image is shown in Figure 1. Additionally, 3 wt% bulk aluminum was added to each nanocomposite. The AZ31 nanocomposites were fabricated using a gravitating stir casting method, which was previously described [4,6]. SiC contents of 0.2 wt%, 0.5 wt% and 1 wt% were added to the cast and the obtained nanocomposites were named AZ31-0.2SiC, AZ31-0.5SiC, and AZ31-1SiC, respectively.

First, the fabricated nanocomposites were polished with different waterproof SiC abrasive papers. Then, their microstructures and elemental analyses were analyzed using SEM and energy dispersive spectroscopy (EDS), respectively. An SEM MAIA3 (Tescan, Brno, Czech Republic) equipped with EDS (Oxford Instruments, Abingdon, UK) was used in this study.

**Figure 2:**
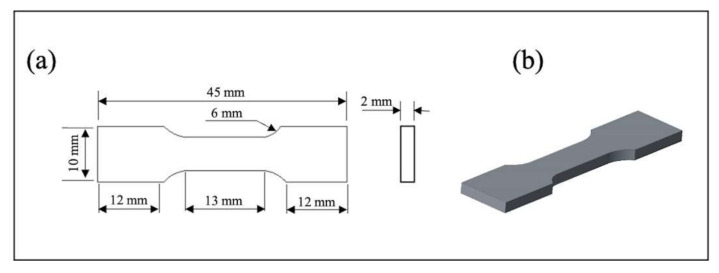
Tensile test specimen (**a**) dimensions and (**b**) schematics.

**Figure 3:**
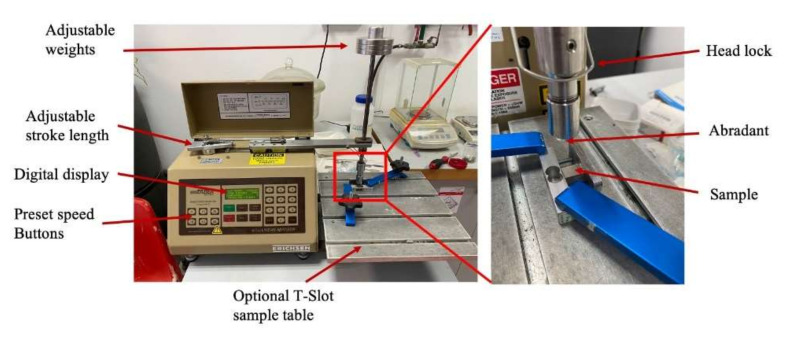
Wear resistance test experimental setup.

Mechanical properties were determined using tensile tests, hardness measurements, and wear resistance. Tensile tests were performed according to the ASTM-E8-69 standard using a universal tensile testing machine, Insight-100 (MTS systems corp., Eden Prairie, MN, USA), with a crosshead speed of 0.5 mm/min. The tensile test specimen dimensions and schematics of the specimens are shown in Figure 2. Prior to tests, all specimens were subjected to polishing with fine abrasive paper to avoid micro-crack induction from the machining process. Vickers hardness was measured using an FV-810 tester (Future Tech, Kawasaki-City, Japan) with a load of 10 kgf for 10 s. The average of five measurements was used in this work. Wear resistance tests were performed in dry conditions using a linear abraser 5750 (Taber Industries, North Tanawanda, NY, USA) with a Si_3_N_4_ ball with a diameter of ¼ in, as shown in Figure 3. Tests were carried out for a total sliding distance of 25.4 m at a speed of 75 cycles/min with a load of 750 g. The weight loss was measured using an Ion BM-5 microbalance (A&D weighting Co., San Jose, CA, USA) with an accuracy of 0.001 mg and an average of three measurements is presented. Examinations of the worn surface were conducted using an RH-2000 digital microscope (Hirox Co., Tokyo, Japan).

## 3. Results and Discussion

### 3.1. Microstructure Evaluation

**Figure 4:**
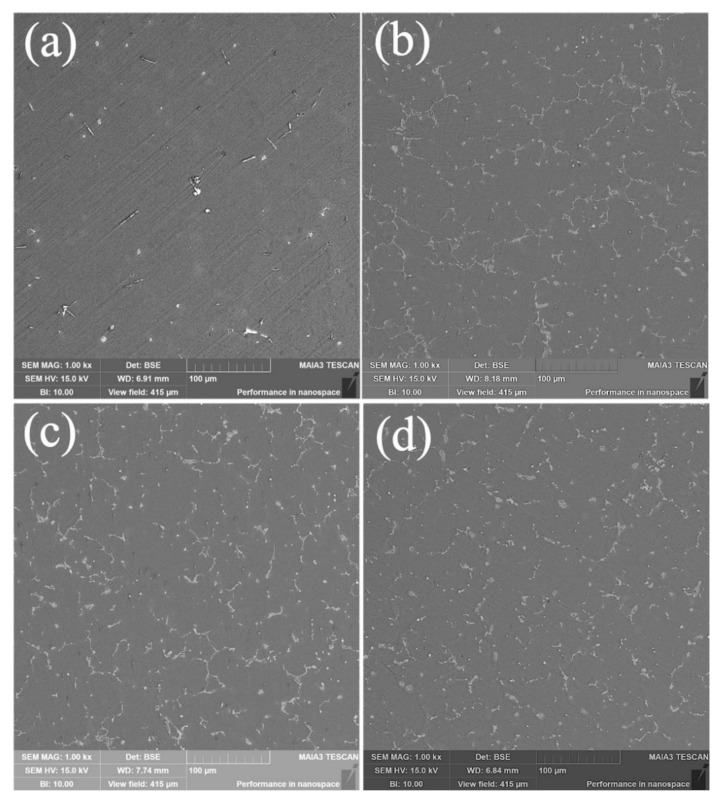
SEM microstructures of (**a**) AZ31, (**b**) AZ31-0.2SiC, (**c**) AZ31-0.5SiC, (**d**) AZ31-1SiC composites.

**Figure 5:**
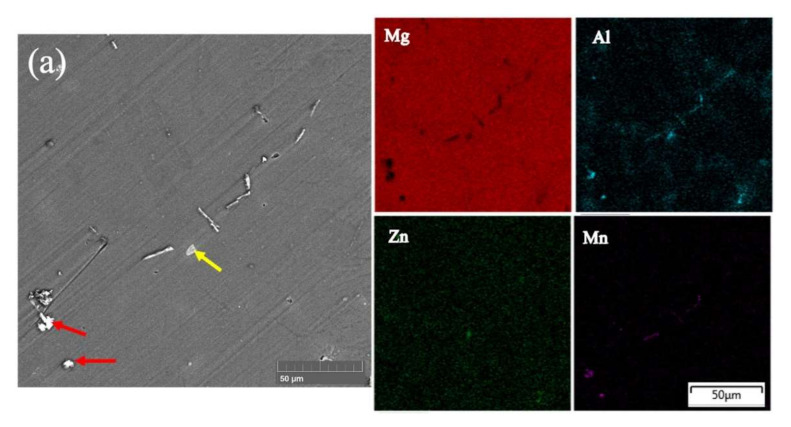

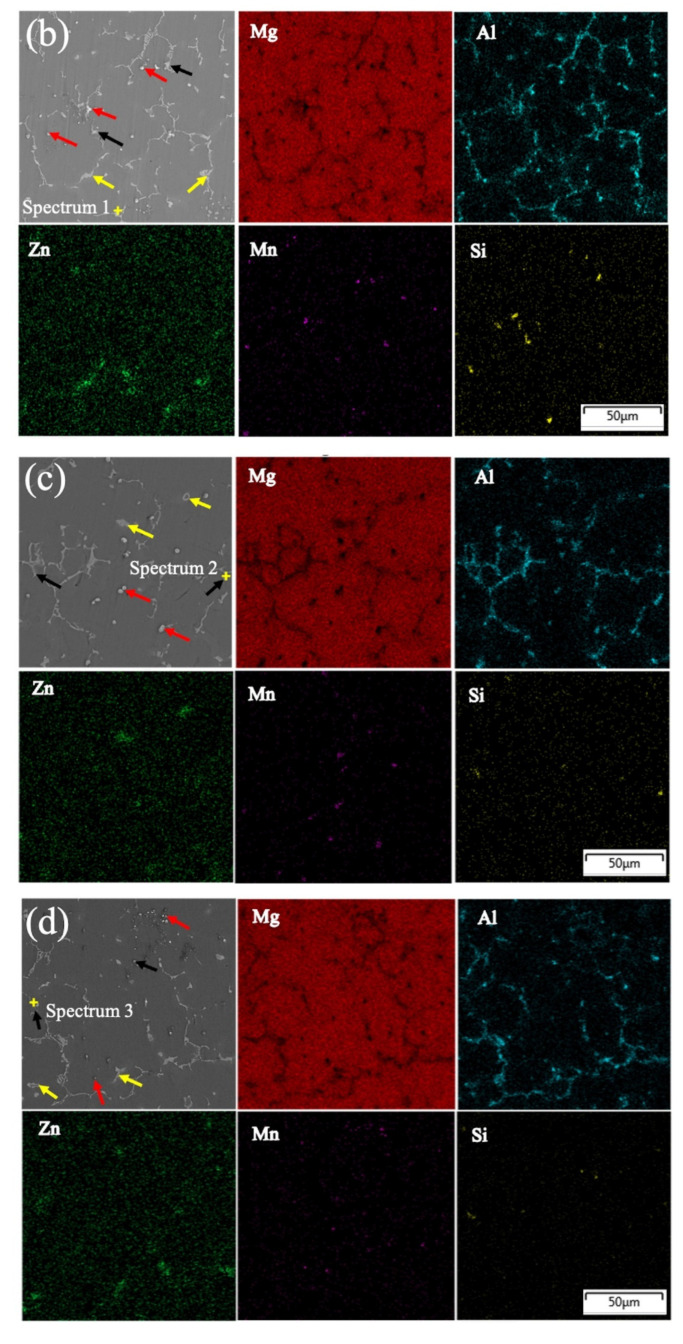
SEM microstructures followed by elemental mapping analysis of (**a**) AZ31, (**b**) AZ31-0.2SiC, (**c**) AZ31-0.5SiC, (**d**) AZ31-1SiC composites. Red arrows point to the Al-Mn phase, yellow arrows point to Mg-Al-Zn, and black arrows point to SiC clusters.

The microstructures of the fabricated AZ31-SiC nanocomposites and AZ31 alloys evaluated using SEM are shown in Figure 4 and Figure 5. It can be seen that the unreinforced AZ31 magnesium alloy microstructure contains a primary Mg phase and traces known to be a Mg_17_Al_12_ phase, which is shown as the white areas in Figure 4a and Figure 5a. However, the fabricated nanocomposite microstructures (Figure 4b–d) show a larger content of the definite discontinuous Mg_17_Al_12_ phase. The increase in content of this intermetallic phase and its homogeneous distribution may be attributed to aluminum dissolution in the metallic matrix, as was also reported in [21]. Sunil et al.’s work also supports the obtained results, and they reported that, when aluminum content in a Mg matrix exceeds 1%, it promotes the formation of an intermetallic phase [22]. No significant changes were detected between all the nanocomposites.

**Figure 6:**
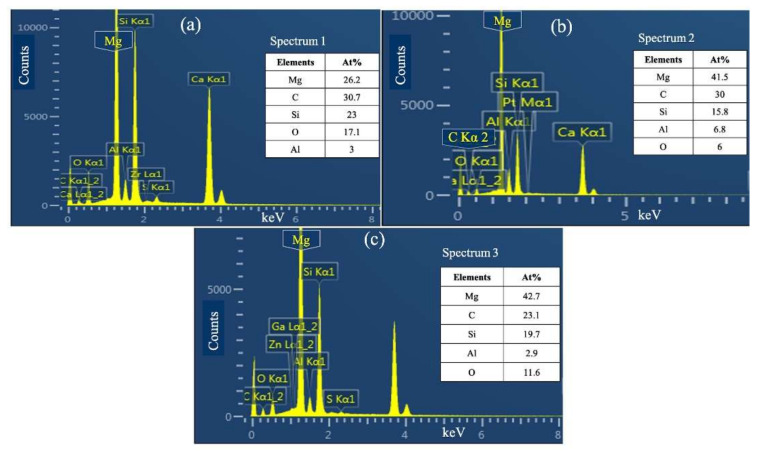
EDS point analysis of (**a**) spectrum 1, shown in Figure 5b, (**b**) spectrum 2, shown in Figure 5c, and (**c**) spectrum 3, shown in Figure 5d.

The elemental mapping analysis shown in Figure 5 confirmed the presence of a Mg_17_Al_12_ intermetallic phase, which was more homogeneously distributed in the AZ31-SiC nanocomposite. This phase was detected as a discontinues network in the Mg matrix. Moreover, appearance of additional Al-Mn and Mg-Al-Zn phases were also detected in the microstructures. The Al-Mn and Mg-Al-Zn phases are indicated by red and yellow arrows on Figure 5, respectively. It was clear that these phases appeared in the nanocomposites and may be due to the addition of Al during fabrication processing. Elemental analyses also pointed to the presence of Si in all nanocomposites, which appeared as the results of SiC nanoparticle addition during fabrication. The map of Si in Figure 5b–d shows homogeneous Si distribution along the metallic matrix. The appearance of SiC in the magnesium matrix was also confirmed by EDS point analysis, shown in Figure 6. Figure 6a–c show that EDS elemental detection was performed on selected points, and were determined as spectrum 1, spectrum 2, and spectrum 3, in Figure 5b–d. Elemental mapping and point analysis jointly confirmed a homogeneous distribution of SiC nanoparticles in nanocomposites, which may be attributed to the stir-casting process, which is an effective method for mixing uniformly.

### 3.2. Mechanical Properties

**Figure 7:**
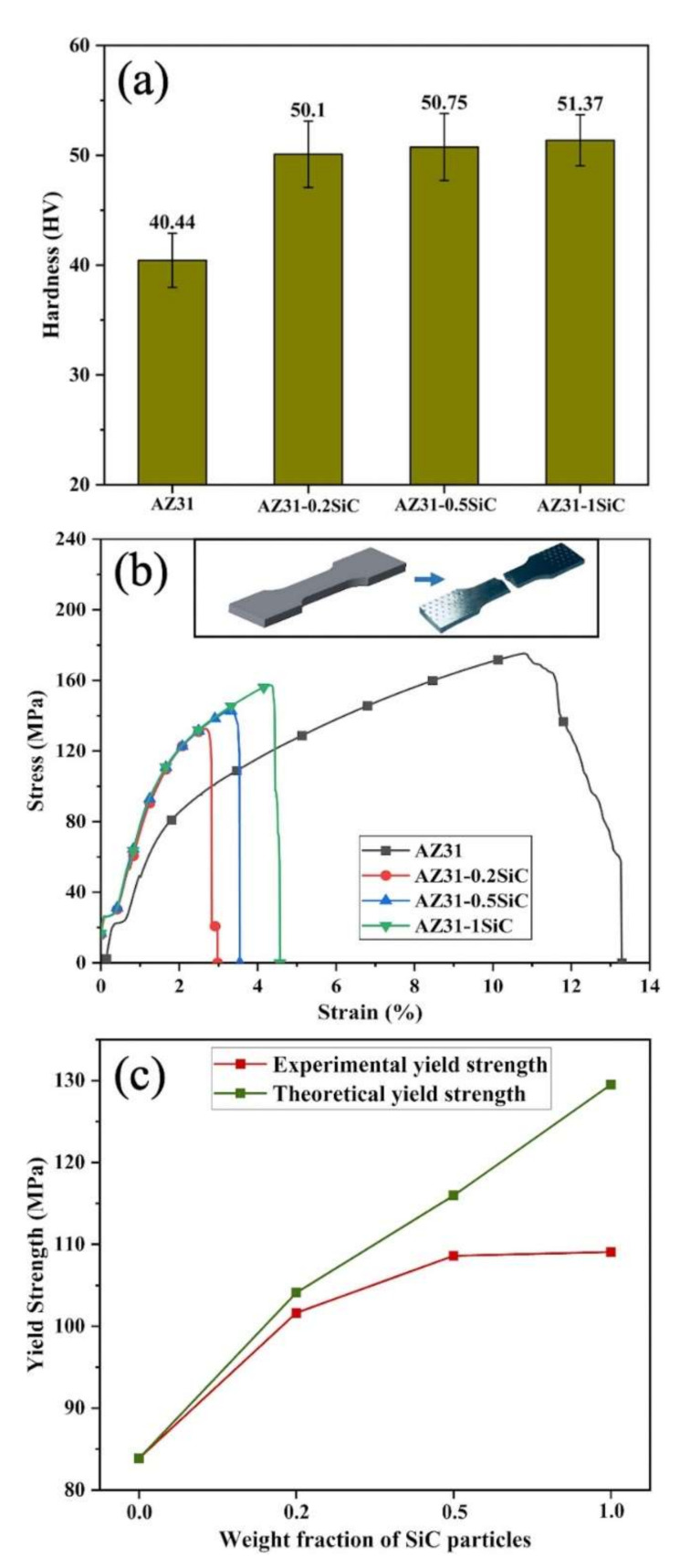
Mechanical properties of AZ31-SiC nanocomposite compared with AZ31 alloy. (**a**) Hardness, (**b**) stress–strain curves, and (**c**) experimental and theoretical yield strengths as a function of SiC nanoparticle content.

The experimental results of the mechanical property measurements are shown in Figure 7. The hardness measurement results are shown in Figure 7a, and revealed that the incorporation of SiC nanoparticles into the AZ31 magnesium alloy matrix significantly affected nanocomposite hardness. Thus, the hardness was gradually increased from 50.1, 50.75, to 51.37 HV, for AZ31-0.2SiC, AZ31-0.5SiC, and AZ31-1SiC, respectively. The hardness of the nanocomposites was more than 25% higher compared to AZ31 alloy, which had a hardness value of 40.44 HV. The enhancement to hardness may be attributed to the existence of hard SiC nanoparticles in the metallic matrix, which restricts highly localized plastic deformation during the indentation test.

The obtained engineering stress–strain curves for the fabricated nanocomposites and the AZ31 alloys are shown in Figure 7b. The values of ultimate tensile strength (UTS), yield stress (YS), and elongation are listed in Table 1. As expected, the incorporation of SiC nanoparticles enhanced the YS of the nanocomposites with an increase in SiC nanoparticles content. The highest enhancement of the YS, by 30%, was detected for AZ31-1SiC nanocomposite compared to the AZ31 alloy.

The hardness and yield strength of the fabricated nanocomposites were enhanced due to the basic strengthening mechanisms in MMCs. These include the coefficient of thermal expansion (CTE), elastic modulus, Orowan strengthening, and load transfer mechanisms [6,23]. Based on previous research, the theoretical strength of the composites, using different statistical approaches, was predicted [6,7,24]. The yield strength of the material is stress-correlated with the dislocation motion. The CTE values of the AZ31 and SiC nanoparticles were $25\times 10^{-6}K^{-1}$ and $6.6\times 10^{-6}K^{-1}$, respectively. This substantial difference between the CTE values of the matrix and reinforcement provided large stresses in the reinforcement–matrix interface. Moreover, these stresses are more valuable for nano-sized reinforcement because of their large surface areas, which may generate the geometrically necessary dislocations (GND) in the vicinity of the matrix. Consequently, CTE mismatch acts as a barrier for the dislocation movement in the metallic matrix, which enhances the overall composite strength. The improvement in the yield strength of the composites due to the matrix–reinforcement CTE mismatch ($\alpha_{CTE}$) can be calculated using Equation (1) [25]:

(1) $$\alpha_{\mathrm{CTE}}=A\times M\times G\times b\times \sqrt{\frac{12\sqrt{2}\times \Delta \alpha_{c}\times \Delta T\times V_{r}}{b\times d_{r}\times \left(1-V_{r}\right)}}$$

where A is the constant characterizing the transparency of the dislocation forest for basal–basal dislocation interactions in magnesium alloy; M is the Taylor factor; G is the shear modulus of the matrix; b is the Burger vector of the matrix; $\Delta \alpha_{c}$ is the CTE difference between the matrix and reinforcement; ΔT is the temperature difference between the process and test; and $V_{r}$ and $d_{r}$ are volume fractions and diameter of the reinforcement.

In a similar manner, the existence of a large difference in the elastic modulus between the matrix and reinforcement also results in a GND interface of the matrix and reinforcement particles. The yield strength changes due to the elastic modulus mismatch ($\Delta \alpha_{EM}$) can be expressed using Equation (2) [24]:

(2) $$\Delta \alpha_{EM}=A\times M\times G\times b\times \sqrt{\frac{6\times V_{r}\times \varepsilon }{b\times d_{r}}}$$

where ε is the elastic strain at the yield. The reinforcement particles influenced the motion of GND in the composite matrix.

The Orowan mechanism has been offered as explanation for the effect of reinforcement under different loading processes. During tensile loading, the movement of dislocations can be inhibited by the reinforcement particles, resulting in the formation of an Orowan loop around these reinforcements, which provides an enhancement to composite strength. The yield strength increase attributed to Orowan strengthening ($\Delta \sigma_{OL}$) can be estimated using Equation (3) [25]:

(3) $$\Delta \sigma_{OL}=\frac{0.4\times M\times G\times b\times \ln\;\left(\frac{d_{r}}{b}\right)}{\pi \times d_{r}\times \left(\sqrt{\frac{4}{\pi \times V_{r}}}-1\right)\times \sqrt{\left(1-ʋ\right)}}$$

where ʋ is a Poisson’s ratio of the metallic matrix.

The load transfer mechanism is another feasible method to improve the strength of the composites. During the loading process, the shear load is transferred from the metallic matrix to the hard reinforcement particles due to the high interfacial bonding between the matrix and reinforcement. Therefore, more load is required to plastically deform the reinforcement particles, which causes an enhancement in composite strength. The change of yield strength due to the load transfer mechanism ($\Delta \sigma_{LT}$) can be expressed using Equation (4) [23,25]:

(4) $$\Delta \sigma_{LT}=\frac{V_{r}\times \sigma_{MYS}}{2}$$

where $\sigma_{MYS}$ is the yield strength of the metallic matrix.

Calculations of the theoretical yield strength were performed using the values listed in Table 2. The above-stated different strengthening mechanisms’ joint contribution was evaluated with the following summarized equation [24]:

(5) $$\sigma_{CYS}=\sigma_{MYS}+\sqrt{\left[{\left(\Delta \alpha_{\mathrm{CTE}}\right)}^{2}+{\left(\Delta \alpha_{EM}\right)}^{2}+{\left(\Delta \sigma_{OL}\right)}^{2}+{\left(\Delta \sigma_{LT}\right)}^{2}\right]}$$

The experimental and theoretical yield strengths of the fabricated nanocomposites are plotted in Figure 7c. It can be seen that both curves have an increasing trend, which correlated with the increase in nano-reinforcement content. Calculations point to the theoretical yield strengths being higher than the experimental yield strengths. These changes may be due to the calculations that were evaluated based on simplifying assumptions and approximations.

The UTS and elongation of AZ31-SiC nanocomposites exhibited lower values compared to the AZ31 alloy. This behavior may be due to the SiC nanoparticles clustering and forming a high-volume fraction of secondary phases at the grain boundaries, which possibly serve as micro-crack initiation sites. The initiated micro-cracks can immediately propagate along with the network of the grain boundaries, which leads to UTS and elongation reductions [27]. However, within nanocomposites, the higher the SiC nanoparticle content the higher the UTS and elongation values. Thus, the determined UTS values were 132.6, 143.1 and 157.2 MPa, and the elongation values were 2.97, 3.54 and 4.56% for AZ31-0.2SiC, AZ31-0.5SiC, and AZ31-1SiC, respectively. This behavior may be attributed to the above-stated joint-strengthening mechanisms.

### 3.3. Wear Behavior

**Figure 8:**
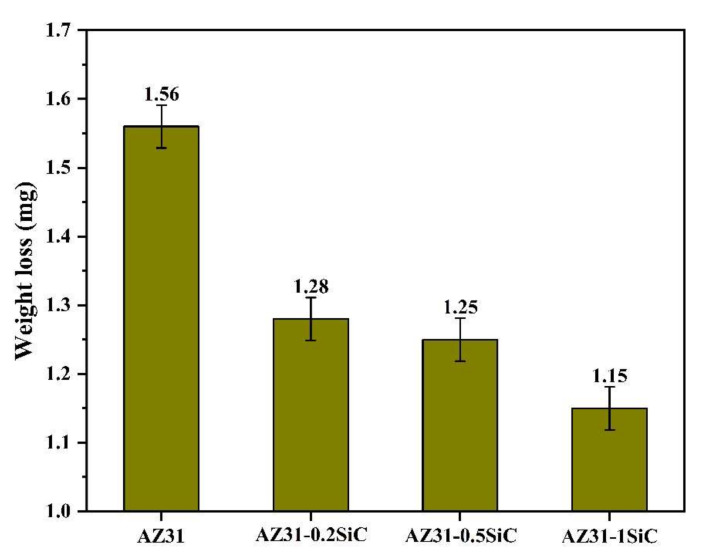
Wear tests results of a weight mass loss of the fabricated AZ31-SiC nanocomposites and AZ31 alloy.

**Figure 9:**
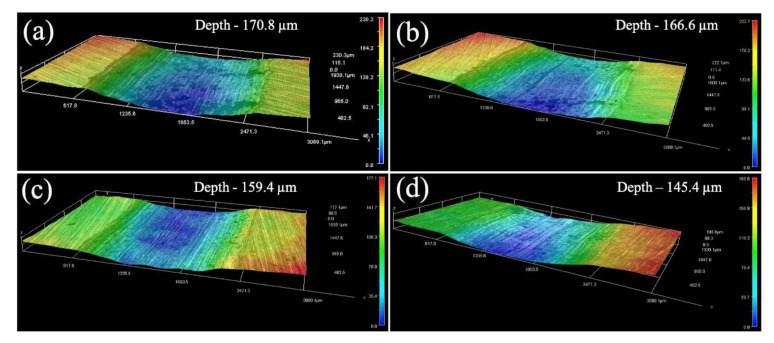
Three-dimensional microscope images of the worn surface of (**a**) AZ31 alloy, and nanocomposites (**b**) AZ31-0.2SiC, (**c**) AZ31-0.5SiC, and (**d**) AZ31-1SiC.

The wear behavior and worn surface images of the fabricated nanocomposites are shown in Figure 8 and Figure 9. As expected, the incorporation of SiC nanoparticles increased the wear resistance of the AZ31-SiC nanocomposites. A reduction in the materials’ weight and a decrease in the penetration depth of the worn surface, were detected. Further, increasing the content of SiC nanoparticles gradually decreased the weight loss, as well as the penetration depth, of the worn surface of the nanocomposites. This behavior was attributed to the appearance of hard SiC nanoparticles in the metallic matrix, which resisted the penetration and cutting of the abrasive material. Furthermore, it is worth adding that the size of the ceramic reinforcement has an important role in the wear behavior of MMCs. Thus, nanoparticles usually result in higher wear resistance compared with micro-sized particles [28]. Study of the wear tests showed mass loss values of 1.28, 1.25, and 1.15 mg for AZ-0.2SiC, AZ-0.5SiC, and AZ-1SiC, respectively, and was less than 1.56 mg for AZ31 alloy.

The 3D microscope images shown in Figure 9 show the worn surfaces obtained for the fabricated nanocomposites and AZ31 alloy. It is indicated that the increase in the content of SiC nanoparticles gradually decreases the depth of the worn surface. Thus, the penetration depth was 166.6, 159.4, and 145.4 µm for AZ-0.2SiC, AZ-0.5SiC, and AZ-1SiC, respectively, and was less than 170.8 µm for AZ31 alloy. The obtained wear behavior is supported by Archard’s well-known work [29]. Archard determined the inverse proportion between the wear weight loss and the hardness of the metallic matrix of soft materials, such as Mg and its alloys.

Evaluation of the worn surface revealed the appearance of many grooves along the sliding direction; therefore, the most suitable wear mechanism for AZ31-SiC nanocomposites is abrasion. In the abrasion wear mechanism, the weight loss of the material occurs due to the sliding under the load, and the nature of the reinforcement is a dominant parameter, e.g., the harder the reinforcement the higher wear resistance of the composite. Moreover, Chattopadhyay revealed that the size of a reinforcement also has an influence on the wear resistance of the composite [30]. He reported that particles less than 1 µm induce sliding, which is prevalent over cutting. This observation fits results obtained in the current work, which demonstrated less material removal with an increase in SiC nanoparticles content.

## 4. Conclusions

AZ31 nanocomposites were successfully fabricated using the stir casting method by the addition of bulk aluminum and SiC nanoparticles as reinforcements. The following conclusions can be derived from the obtained results:

1. The microstructure of Al-SiC nanocomposites showed the existence of discontinuous Mg_17_Al_12_ phases and a uniform distribution of SiC nanoparticles that affect the mechanical properties of nanocomposites.
2. The hardness and YS strengths were enhanced with the increase in SiC nanoparticle content. The maximum obtained values were 51.37 HV and 109.07 MPa, respectively, which were attained for the AZ31-1SiC nanocomposite. The enhancement of the hardness and YS was attributed to the joint contribution of the CTE, elastic modulus, and the Orowan and load-transfer strengthening mechanisms.
3. Wear tests revealed a decrease in weight loss with an increase in SiC content in AZ31-SiC nanocomposites. This behavior was also confirmed by 3D images of the worn surface. The fabricated nanocomposites showed wear behavior that was inversely proportional to the hardness, which was attributed to the abrasion wear mechanism.

## Figures and Tables

**Table 1:** Mechanical properties values for the fabricated AZ31-SiC nanocomposites and AZ31 alloy.

Material	YS (MPa)	UTS (MPa)	Elongation (%)	Hardness (HV)	Theoretical YS (MPa)
AZ31	83.87	175.3	13.287	40.44	-
AZ31-0.2SiC	101.63	132.6	2.974	50.1	104.02
AZ31-0.5SiC	108.61	143.1	3.54	50.75	115.86
AZ31-1SiC	109.07	157.2	4.56	51.37	129.35

**Table 2:** Values of the theoretical yield strength parameters used in the calculation of Equation (5) [6,24,25,26].

*A*	*M*	*G* (GPA)	*b* (nm)	*d_r_* (nm)	Δ*α_c_* (K^−1^)	Δ*T* (K)	*ε*	ʋ	σ_*MYS*_ (MPa)
0.25	3.8	17.3	0.325	60	1.84 × 10^−5^	300	0.019	0.281	83.878

## Data Availability

The data presented in this study are available on request from the corresponding author.
